# High-throughput screening identification of apigenin that reverses the colistin resistance of mcr-1-positive pathogens

**DOI:** 10.1128/spectrum.00341-24

**Published:** 2024-09-09

**Authors:** Feng Tang, Wenjing Peng, Xu Kou, Zeliang Chen, Libo Zhang

**Affiliations:** 1 College of Animal Science and Veterinary Medicine, Collaborative Innovation Center for Prevention and Control of Zoonoses, Jinzhou Medical University, Jinzhou, Liaoning, China; Seton Hall University, South Orange, New Jersey, USA

**Keywords:** apigenin, mcr-1, colistin resistance, antimicrobial, infection

## Abstract

The plasmid-mediated gene mcr-1 that makes bacteria resistant to the antibiotic colistin is spreading quickly, which means that colistin is no longer working well to treat Gram-negative bacterial infections. Herein, we utilized a computer-aided high-throughput screening drugs method to identify the natural product apigenin, a potential *mcr*-protein inhibitor, which effectively enhanced the antimicrobial activity of colistin. Several assays, including a checkerboard minimum inhibitory concentration assay, a time-kill assay, the combined disk test, molecular simulation dynamics, and animal infection models assay, were conducted to verify whether apigenin enhanced the ability of colistin to fight Gram-negative bacterial infections. The results showed that apigenin improved the antimicrobial activity of colistin against multidrug-resistant *Enterobacteriaceae* infection. Moreover, apigenin not only did not increase the toxic effect of colistin but also had the ability to effectively inhibit the frequency of bacterial resistance mutations to colistin. Studies clearly elucidated that apigenin could interfere with the thermal stability of the protein by binding to the *mcr*-1 protein. Additionally, the combination of apigenin and colistin could exert multiple effects, including disrupting bacterial membranes, the generation of bacterial nitric oxide and reactive oxygen species, as well as inhibiting bacterial adenosine triphosphate production. Furthermore, the addition of apigenin was able to significantly inhibit colistin-stimulated high expression levels of the bacterial mcr-1 gene. Finally, apigenin exhibited a characteristic anti-inflammatory effect while enhancing the antimicrobial activity of colistin against mcr-1-positive *Escherichia coli* (*E. coli*) infected animals. In conclusion, as a potential lead compound, apigenin is promising in combination with colistin in the future treatment of mcr-1-positive *E. coli* infections.

**IMPORTANCE:**

This study found that apigenin was able to inhibit the activity of the *mcr*-1 protein using a high-throughput virtual screening method. Apigenin effectively enhanced the antimicrobial activity of colistin against multidrug-resistant *Enterobacteriaceae*, including mcr-1-positive strains, *in vitro* and *in vivo*. This study will provide new options and strategies for the future treatment of multidrug-resistant pathogen infections.

## INTRODUCTION

In recent years, antimicrobial resistance (AMR), as a major threat to human health, has caused global economic losses and a public health crisis (1). The development of AMR seriously compromises modern medical treatment for bacterial infections, especially related infectious diseases caused by Gram-negative bacteria (2, 3). Gram-negative bacteria have many defense strategies, including highly impermeable outer membrane barrier, robust efflux system, and formation of dense biofilms, which were further complicated by the widespread and irrational use of antibiotics (4–6). The worldwide spread of multidrug-resistant (MDR) Gram-negative bacteria and the mobile colistin resistance (mcr) gene has made antimicrobial chemotherapy difficult, making most antibiotics, including colistin, ineffective in treating bacterial infections (7–9). The D-ornithine (Dab) residue in colistin carries a positive charge and has electrostatic interaction with the negatively charged phosphate residue in lipid A, thereby increasing the permeability of the bacterial membrane, resulting in the leakage of cytoplasmic contents and causing the death of bacteria. The *mcr*-1 protein exhibits phosphoethanolamine (PEA) transferase activity, catalyzing the transfer of PEA onto lipid A, thereby reducing the electronegativity of lipid A (7). This alteration affects the interaction between colistin and bacterial outer membranes, resulting in bacterial resistance to colistin. Adding to the urgency of the situation, the new antibiotics approved over the past two decades are also not addressing the problem (10, 11). Therefore, alternative strategies are urgently needed to solve this problem influencing the whole world.

In the past, six new antibiotics, including linezolid, daptomycin, ceftaroline, ceftobiprole, delafloxacin, and tedizolid, have been approved but only very few of them can fight against Gram-negative bacteria, which makes it harder to treat infections caused by Gram-negative bacteria (10–12). Compared with the development of new antibiotics, combination therapy is a safer, more economical, and more effective alternative therapy, which has attracted great attention (13, 14). Furthermore, combination treatment has been effectively utilized within the clinical treatment of β-lactam antibiotic-resistant microscopic organisms and accomplished very great healing impact (15, 16). Antibiotic adjuvants are typically co-administered with antibiotics to enhance their therapeutic efficacy or mitigate side effects. These adjuvants can be chemical compounds or natural substances that aid in the more effective penetration of antibiotics into bacterial cells or enhance their pharmacokinetic properties *in vivo* (14). Accordingly, combination therapy or the addition of certain antibiotic adjuvants may be able to re-sensitize MDR bacteria with a resistant phenotype to colistin.

A large number of natural products with antimicrobial activity are expected to become lead compounds to enter the new drug development stage (17, 18). Exploring colistin adjuvants among the wide variety of natural products can be a viable strategy against MDR Gram-negative bacteria. As a method for predicting protein–ligand interactions, virtual screening has several advantages over high-throughput screening, such as screening time, cost, biological activity, and screening effect of special targets. These advantages make virtual screening a powerful potential in the current field of drug development (19). Therefore, we performed a high-throughput virtual screening of a compound library with a capacity of 19,000. Here, we found that apigenin performed the best among the flavonoids in scoring interactions with *mcr*-1 protein. As a flavonoid, apigenin exhibits a variety of biological activities. In addition to its already reported anti-inflammatory activity, apigenin can also act as a therapeutic agent to overcome neurodegenerative diseases, a few types of cancer, as well as viral infections (20–22). Further antimicrobial ability assays and animal test results revealed that apigenin re-sensitized mcr-1-positive strains to colistin *in vitro* and *in vivo*. In addition, the mechanisms of action of the combination of apigenin and colistin was deeply investigated using molecular dynamics simulations assay, molecular interactions assays, and fluorescence detection assays. Collectively, all data indicate that this combination provides a viable therapeutic scheme for the therapy of mcr-1-positive pathogens and represents a new way to address the threat of colistin resistance to a certain extent.

## RESULTS

### High-throughput virtual screening based on *mcr*-1 protein structure

**Fig 1:**
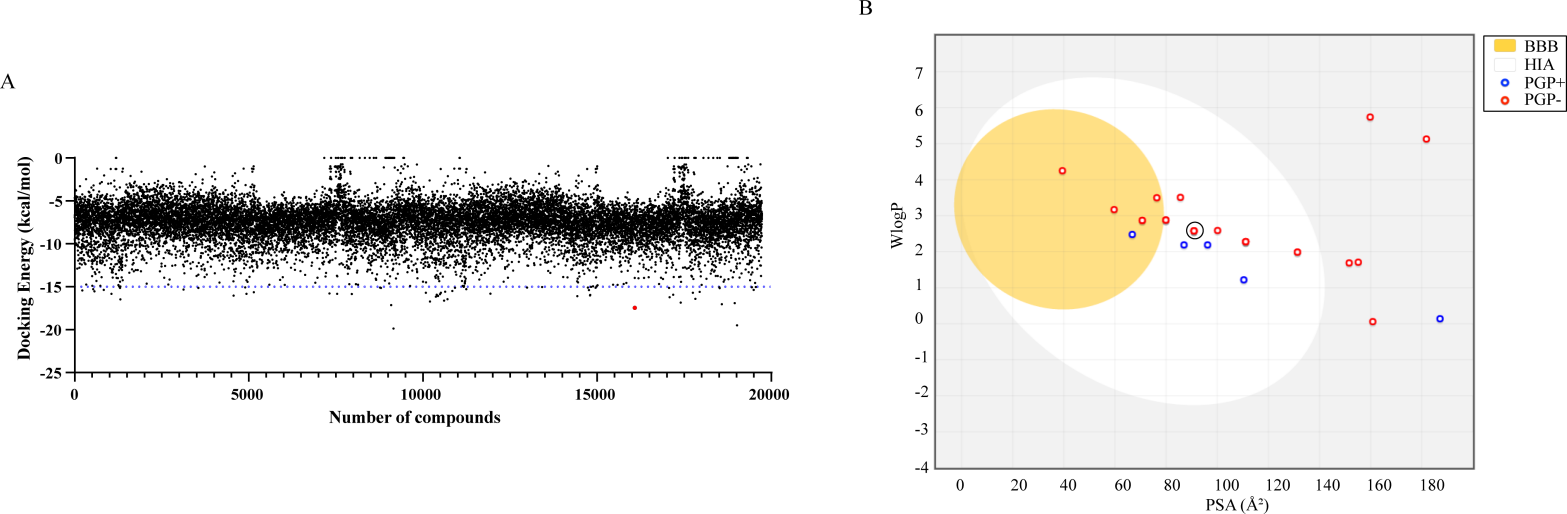
The screening determination of apigenin. (**A**) Docking energy for 19,732 compounds targeting *mcr-1* protein using CADD methods; (**B**) Brain-or-Intestinal Estimated Permeation (BOILED-Egg) diagrams of 30 flavonoids. The yellow area indicates molecules predicted to permeable blood–brain barrier (BBB). Absorbable gastrointestinal (HIA) molecules were marked in a white area. Molecular characterization of the blue region was predicted to permeable glycoprotein substrates (PGP+), while red represented non-substrates (PGP−).

Computer-Aided Drug Design (CADD) has gotten to be a commonly used tool in drug discovery and development. Computational chemistry-based approaches to find lead compounds have been widely used by modeling and calculating the relationship between drugs and receptors (23). References are to the catalytic structural domain of the *mcr*-1 protein (24), whose crystal structure (Protein Data Bank: 5GRR) was used to screen more than 19,000 compounds (Fig. 1A). Each compound was bound to the protein in a different conformation by means of semi-flexible docking, and the affinity energy between each conformation and the protein was obtained. Ranking of the compounds based on the docking energy revealed that the flavonoid apigenin was among the top 10 compounds (Table S1), given that flavonoids, such as honokiol, phloretin, and naringenin, have been reported to have potential as *mcr*-1 protein inhibitors. Moreover, apigenin had the lowest docking energy value among all the flavonoids screened (Fig. S1). After excluding some acids and alcohols compounds, we finally identified apigenin as a potential mcr-1 inhibitor. Although some flavonoids have been reported to inhibit *mcr*-1 protein activity, the specific effects and mechanisms of action of apigenin remain unclear. Furthermore, physicochemical parameters of 30 flavonoids were calculated using the online SwissADME tool, which revealed that apigenin had good physicochemical properties and could be absorbed in the gastrointestinal tract (Fig. 1B). All the data suggested that apigenin was more promising as a potential candidate for drug screening programs of *mcr*-1 inhibitors compared to similar compounds.

### Apigenin re-sensitized mcr-1-positive strains to colistin *in vitro*

**Fig 2:**
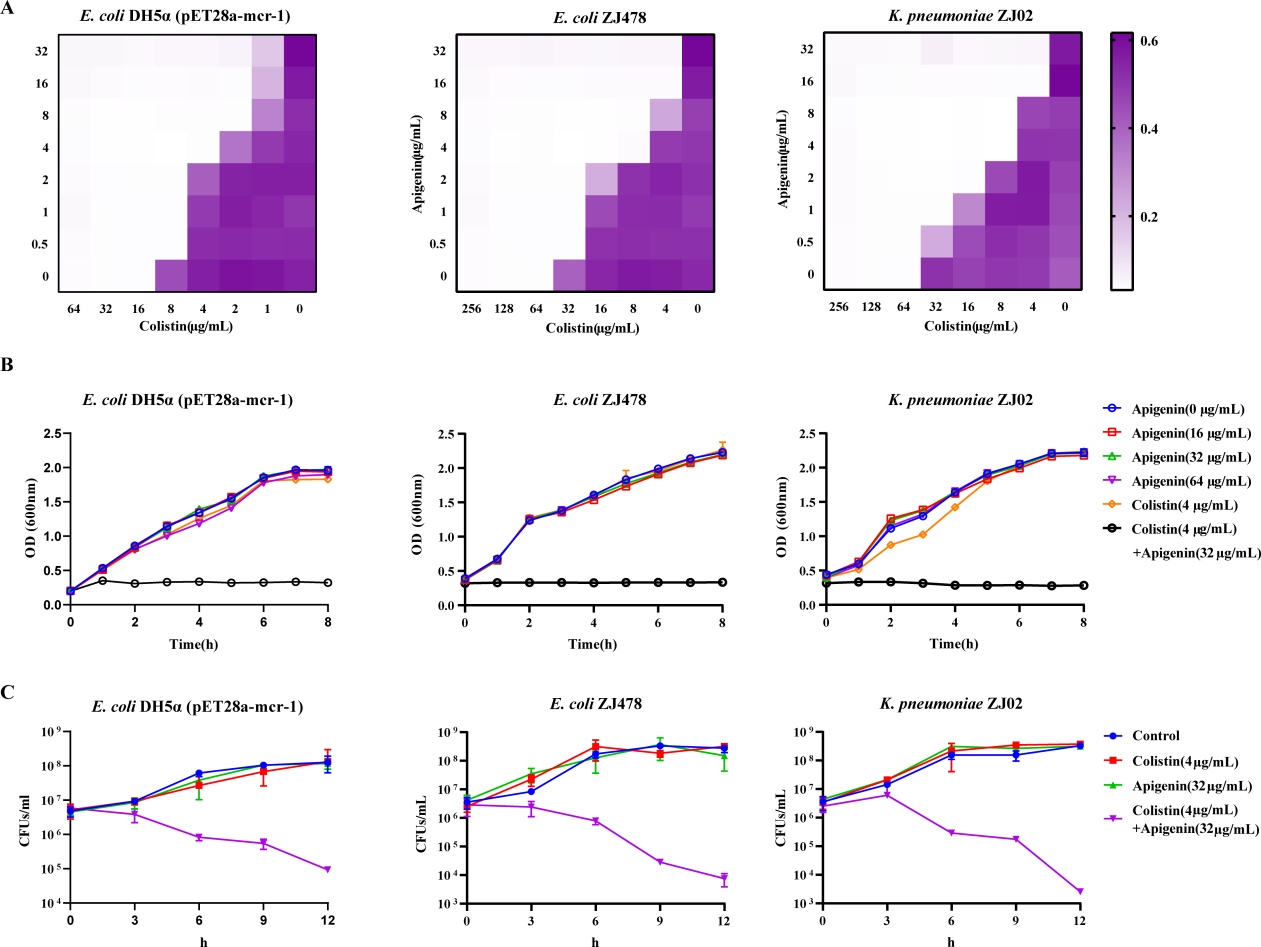
Apigenin reversed the drug resistance of mcr-1-positive bacteria to colistin. (**A**) The combined effect of the apigenin and colistin on mcr-1-positive strains (*E. coli* DH5α, *E. coli* ZJ478, and *K. pneumoniae* ZJ02, respectively) appeared using microdilution checkerboard analysis; (**B**) the effect of drugs on growth curves of mcr-1-positive strains (*E. coli* DH5α, *E. coli* ZJ478, and *K. pneumoniae* ZJ02, respectively); (**C**) kill curves of mcr-1-positive strains (*E. coli* DH5α, *E. coli* ZJ478, and *K. pneumoniae* ZJ02, respectively), after treatment with apigenin, colistin, the combination, and the control (no antibiotic) group. Data were obtained from three biological replicates, and the mean ± SD was shown.

To further explore the synergistic effect of apigenin and colistin, we performed the minimum inhibitory concentration (MIC) tests *in vitro*. The checkerboard assay revealed the synergistic effect of the combined regimen of apigenin and colistin on three mcr-1-positive strains, with fractional inhibitory concentration (FIC) index of 0.188, 0.125, and 0.188, respectively (Fig. 2A). Moreover, our results suggested that the combination regimen of apigenin and colistin showed an initial synergistic or additive effect on mcr-1-negative strains, with FIC indices ranging from 0.3 to 0.6 (Table 1), indicating that there may be other mechanisms at work with apigenin. Subsequently, the growth curves revealed that apigenin did not have a negative effect in the growth process of mcr-1-positive *E. coli* or *Klebsiella pneumoniae (K. pneumoniae*) (Fig. 2B). However, apigenin combined with colistin inhibited bacterial growth for all 8 hours of testing, and those strains have been shown to express the mcr-1 gene in the previous reference (25). To further clarify whether the combination of apigenin and colistin was bacteriostatic or bactericidal against mcr-1-positive bacteria, we performed the time-kill curves test. The results showed that apigenin (32 µg/mL) combined with colistin (4 µg/mL) was effective in killing mcr-1-positive bacteria. Apigenin combined with colistin could effectively kill mcr-1-positive strains at 6 to 12 hours (Fig. 2C). In addition, the combined disc test (CDT) showed that apigenin combined with colistin had an effectively larger zone of inhibition against the mcr-1-positive *E. coli* and *K. pneumoniae* strains compared to colistin alone (Fig. S2). Meaningfully, the addition of apigenin effectively reduced the frequency of bacterial resistance mutations to colistin (Fig. S3). The MIC of colistin alone changed 64-fold. However, with the addition of apigenin, the MIC of colistin changed only twofold. In brief, these data suggested that apigenin re-sensitized mcr-1-positive strains to colistin *in vitro*.

**TABLE 1:** MIC values for colistin and FIC values of combination with apigenin

Strain	mcr-1	Apigenin MIC (µg/mL)		Colistin MIC (µg/mL)		FIC index
		Alone	Combination	Alone	Combination	
*E. coli* ATCC25922	−	128	4	0.5	0.125	0.313
*E. coli* CF5-1	−	128	8	0.5	0.25	0.563
*E. coli* CN3-1	−	256	8	1	0.25	0.313
*E. coli* DP10-1	−	128	16	0.5	0.25	0.625
*E. coli* DP1-1	−	128	16	1	0.0625	0.188
*E. coli* DP11-YY	+	128	8	16	0.5	0.09
*E. coli* DP12-YY	+	256	32	32	4	0.25
*E. coli* DP14-1	+	128	16	16	2	0.188
*E. coli* DP14-YY	+	128	8	16	2	0.25
*E. coli* DP14-CY	+	128	16	32	8	0.188

### Apigenin inhibited protein bioactivity by directly binding to *mcr*-1 protein

**Fig 3:**
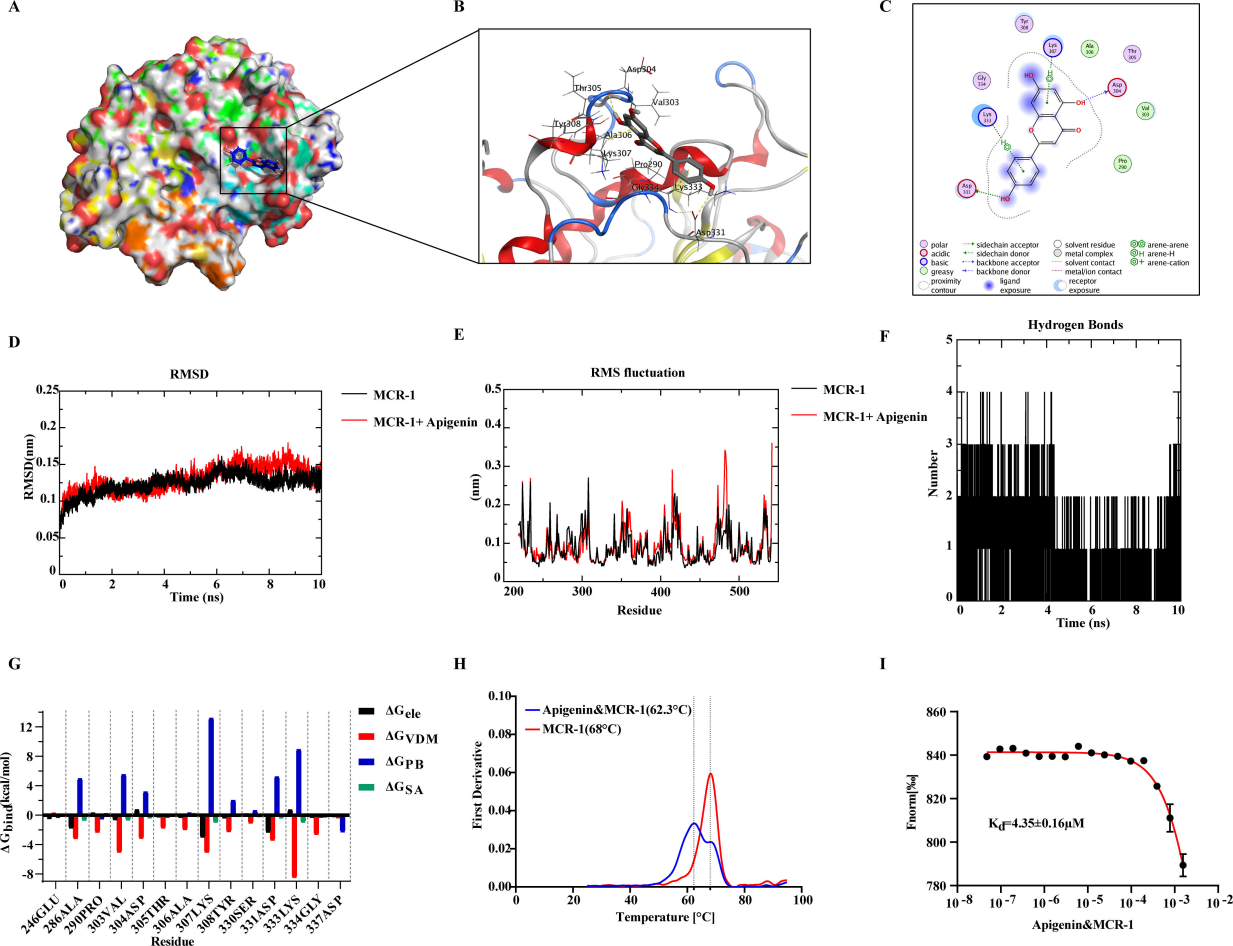
The determination of molecular interactions between apigenin and *mcr*-1 protein. (**A**) Binding pocket of apigenin to *mcr*-1 protein (total view); (**B**) three-dimensional display of the binding site of the complex system; (**C**) two-dimensional display of the binding pocket of the complex system using MOE; (**D**) the RMSD fluctuations of the complex system and only *mcr*-1 in the complex system throughout the simulation; (**E**) RMSF of the complex system and only *mcr*-1 in complex system; (**F**) hydrogen bonding during binding of apigenin to *mcr*-1 protein; (**G**) the binding free energy, calculated by MMPBSA; (**H**) protein thermal stability of *mcr*-1; (**I**) MST binding curves of *mcr*-1 complexed with apigenin (*K*_d_ = 4.35 µM).

To clarify how apigenin inhibited *mcr*-1 protein, we simulated the interaction between apigenin and the protein using molecular dynamics simulations. According to the results of molecular docking using Autodock-based software Smina (version 2020), apigenin could attach to the grooves on the surface of the *mcr*-1 protein (Fig. 3A). The potential binding pockets on the proteins consisted mainly of Pro290, Val303, Asp304, Thr305, Tyr308, Ala306, Lys307, Gly334, Lys333, and Asp331 (Fig. 3B). Interactions between proteins and small molecules were further analyzed using MOE (v. 2009). The interaction between apigenin and the active region of the protein was mainly hydrogen bond, electrostatic force, and van der Waals force (Fig. 3C). Analysis of the root mean square deviation (RMSD) of the *mcr*-1 protein and *mcr*-1-apigenin complexes confirmed that protein and protein complexes reached equilibrium after 2 ns and the curves fluctuated gently and stabilized around 0.125 nm, indicating that the protein conformation did not obviously change after the binding of apigenin to the *mcr*-1 protein (Fig. 3D). Furthermore, the results of root mean square fluctuations (RMSF) were used to characterize the degree of spatial shift of individual amino acid residues. In detail, the results showed that residues 303–308 of the *mcr*-1–apigenin complex showed a weakened trend of the RMSF, which suggested a decrease in the flexibility of these amino acid residues, further reflecting an interaction between these amino acids and apigenin. Higher RMSF values implied greater flexibility of amino acid residues in the protein, indicating a structural change in the protein. Residues 347–355, 412–415, and 471–485 of the *mcr*-1–apigenin complex showed greater flexibility than that of *mcr*-1 protein, indicating that apigenin caused structural changes of the *mcr*-1 protein (Fig. 3E). Moreover, the analysis of the molecular dynamics simulation process also found the presence of hydrogen bonding (Fig. 3F), which was also consistent with the appearance of hydrogen bonding in the molecular docking results (Fig. 3B). Meanwhile, the energy decomposition curves verified that apigenin interacted with *mcr*-1 protein mainly through electrostatic and van der Waals forces (Fig. 3G). In addition, we also determined the binding capacity of apigenin and *mcr*-1 protein *in vitro*. Protein thermal stability experiments revealed that the stability of *mcr*-1 protein changed with the addition of apigenin. The initial unchaining temperature decreased by 7.2°C and the *T*_m_ value decreased by 5.7°C, indicating that the *mcr*-1 protein became more unstable and more sensitive to temperature after binding to apigenin (Fig. 3H). Meanwhile, microscale thermophoresis (MST) experiments further showed that apigenin and *mcr*-1 protein had strong binding with a *K*_d_ value of 4.35 µM (Fig. 3I). In short, all data suggested that apigenin disrupted the activity of the protein by directly binding to the *mcr*-1 protein.

### The combination of apigenin and colistin induced bacterial membrane damage

**Fig 4:**
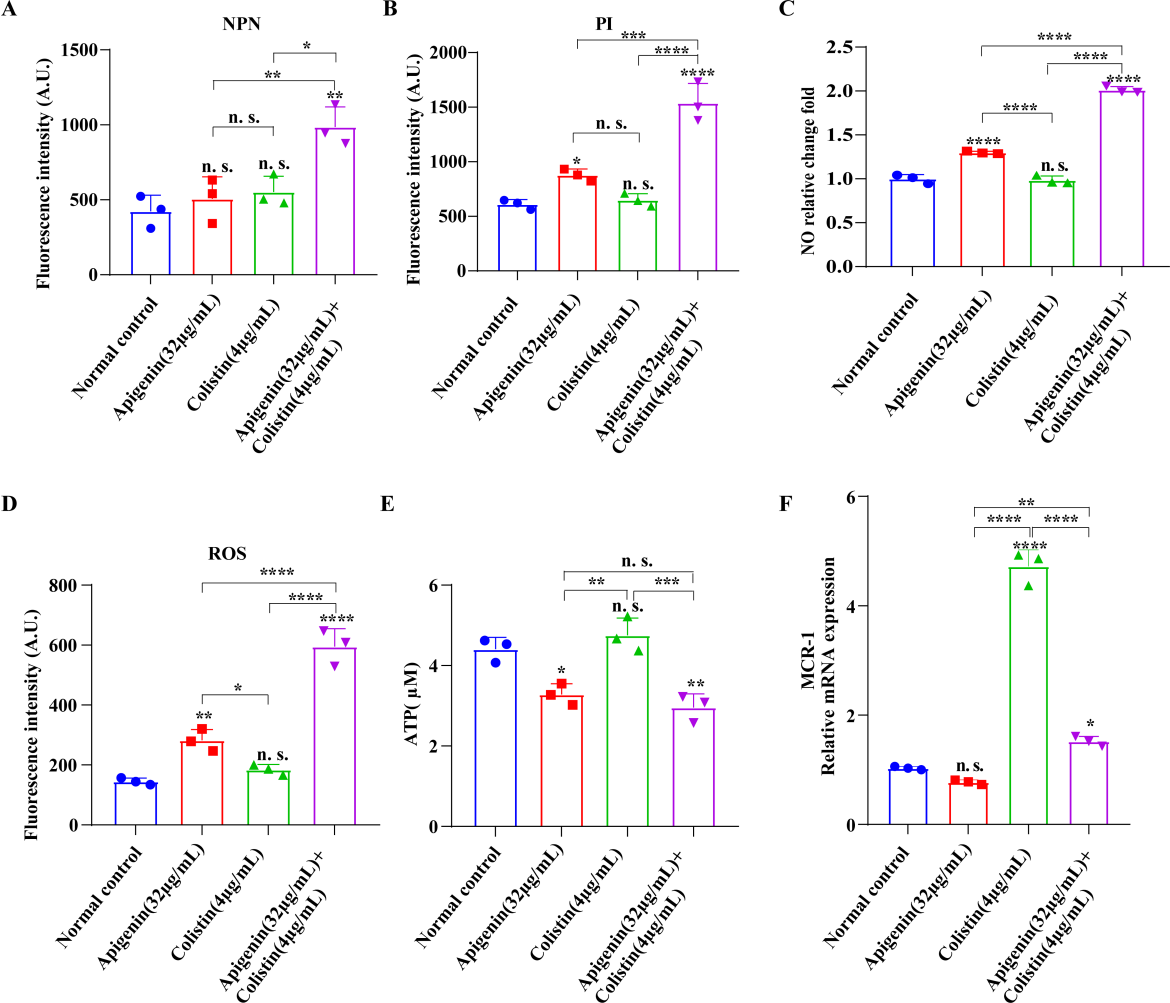
The effect of apigenin combined with colistin on bacterial membrane damage and antibacterial mechanism. (**A**)The damaging effect of the combined protocol of apigenin and colistin on the outer membrane of *E. coli* ZJ478 was characterized by the fluorescence intensity of 1‐N‐phenylnaphthylamine (NPN) staining; (**B**) the damaging effect of the combined protocol of apigenin and colistin on the inner membrane of *E. coli* ZJ478 was characterized by the fluorescence intensity of propidium iodide (PI); (**C**) the bacterial NO production was determined using nitric oxide assay kit; (**D**) the bacterial ROS production was determined. Probed with 2′,7′-dichlorodihydrofluorescein diacetate (DCFH-DA); (**E**) the detection of bacterial intracellular ATP content; (**F**) the transcription of mcr-1 in *E. coli* ZJ478, determined by RT‐PCR analysis. The one-way analysis of variance (ANOVA) with post-hoc Tukey tests was used to calculate *P* values (n. s., not significant; *P < 0.05, **P < 0.01, ***P < 0.001, and ****P < 0.0001).

Considering that the combined strategies of apigenin and colistin had similar synergism or additive effect on mcr-1-negative strains *in vitro*, we hypothesized that other mechanisms of action existed in addition to direct interactions with *mcr*-1 proteins. Therefore, using fluorescence intensity measurement, we first confirmed if apigenin disrupted the permeability of the bacterial cytoplasmic membrane and outer membrane. We found that apigenin had only a slight effect on the bacterial outer membrane, but apigenin combined with colistin greatly increased the permeability of the outer membrane (Fig. 4A). Moreover, apigenin combined with colistin similarly increased bacterial cytoplasmic membrane permeability, and apigenin alone also induced an increase in bacterial cytoplasmic membrane permeability (Fig. 4B). The results of scanning electron microscopy showed that apigenin combined with colistin caused severe wrinkling and rupture of the bacteria compared to the blank control group (bacteria treated with 0.3% DMSO), whereas apigenin alone also showed a milder bacterial wrinkling, which suggested that apigenin had an influence on the bacterial membrane (Fig. S4). Subsequently, we tested the effect of apigenin on bacterial nitric oxide (NO) and reactive oxygen species (ROS) and found that apigenin could induce bacterial NO and ROS production (Fig. 4C and D). However, apigenin did not cause bacterial death at this concentration, as shown by the results of Fig. 2F. We hypothesized that the strong outer membrane structure of Gram-negative bacteria exerted a protective effect on the bacteria but the combination of apigenin colistin caused more bacterial NO and ROS production than that of drugs alone, which in turn led to the death of the bacteria. Meanwhile, assays of bacterial adenosine triphosphate (ATP) showed that apigenin was effective in inhibiting ATP production (Fig. 4E). Furthermore, as demonstrated by the results of fluorescence quantitative detection, colistin could promote high expression of the bacterial mcr-1 gene, whereas the addition of apigenin was able to significantly inhibit colistin-stimulated high expression levels of the bacterial mcr-1 gene (Fig. 4F). In addition, the expression level of mcr-1 gene was significantly decreased in the apigenin combination group compared to the colistin-treated group, suggesting that the addition of apigenin could inhibit the expression of mcr-1 gene and further re-sensitize the bacteria to colistin. Collectively, these data showed that the combination of apigenin and colistin could exert multiple effects, which in turn promoted the antimicrobial ability of colistin.

### Apigenin enhanced the antimicrobial activity of colistin against mcr-1-positive *E. coli* infected animals

**Fig 5:**
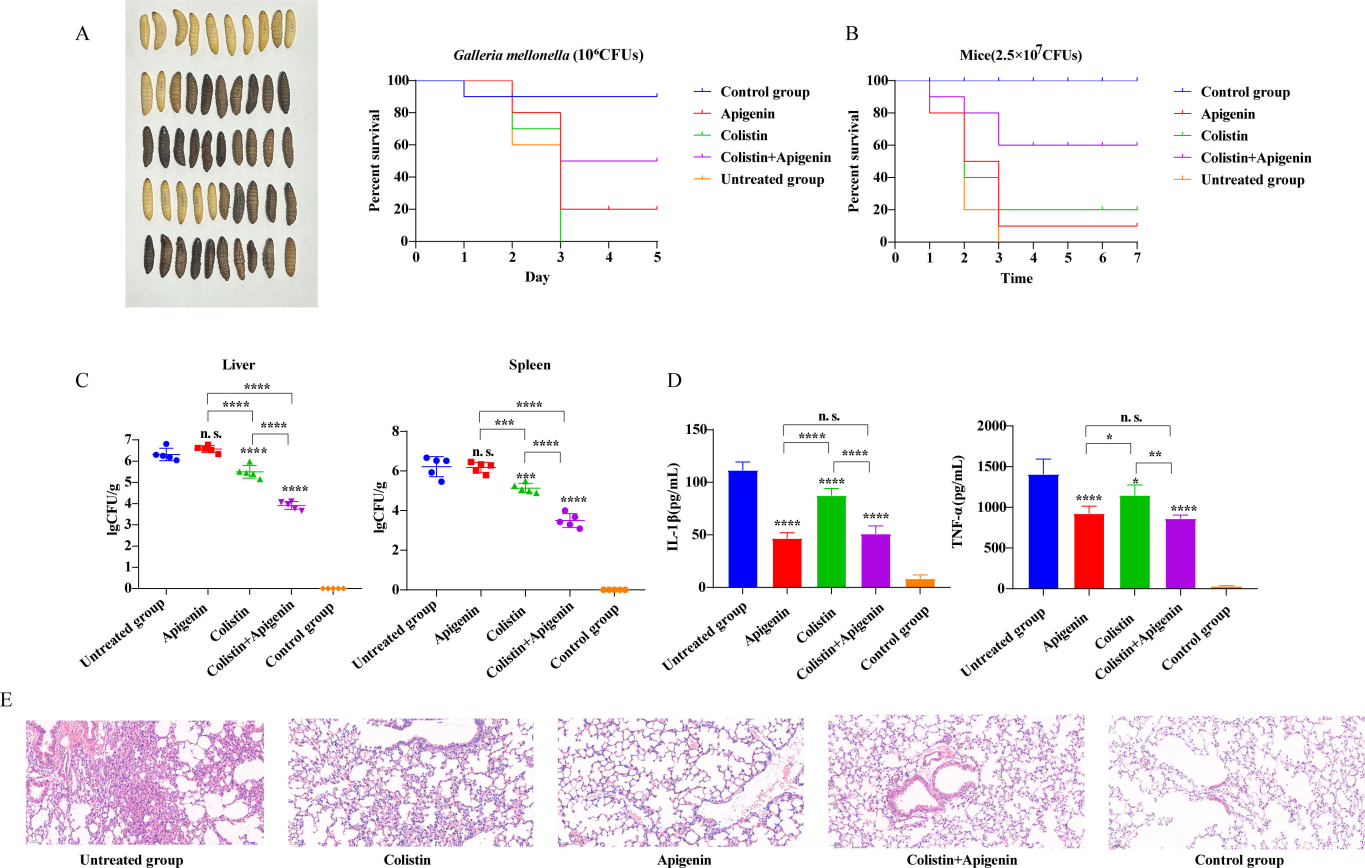
Apigenin enhanced the antimicrobial effect of colistin *in vivo*. (**A**) *G. mellonella* larvae (*n* = 10 per group) survival after infection with *E. coli* ZJ478 (1.0 × 10^6^ CFU). It was divided into five groups: apigenin (2 mg/kg) group, colistin (2 mg/kg) group, combination (2 + 2 mg/kg) group, untreated group (injected with bacteria), and control group (injected with PBS buffer containing 0.8% DMSO); (**B**) survival of female BALB/C mice (*n* = 10 per group) after *E. coli* ZJ478 (2.5 × 10^7^ CFU) infection. Five groups were created: apigenin (10 mg/kg), colistin (5 mg/kg), the combination medication (10 + 5 mg/kg), untreated group (injected with bacteria), and control group (injected with PBS buffer containing 0.5% CMC-Na); (**C**) the bacterial load in the liver and spleen; (**D**) the expression levels of TNF-α and IL-1β in infected mice; (**E**) sections of lung tissue from mice infected with *E. coli* ZJ478 showed pathological alterations. The one-way ANOVA with post-hoc Tukey tests was used to calculate *P* values (n. s. not significant; *P < 0.05, **P < 0.01, ***P < 0.001, and ****P < 0.0001).

Potential toxic effects, including nephrotoxicity and neurotoxicity, are one of the important factors limiting the clinical application of colistin (26). To assess whether apigenin affected colistin toxicity, we first determined the cytotoxicity and hemolysis of the combined protocol of apigenin and colistin *in vitro* prior to experimental animals. Encouragingly, apigenin did not increase the hemolytic activity of colistin on erythrocytes (Fig. S5A). Similarly, no increase in colistin cytotoxicity was observed with the addition of apigenin (Fig. S5B). Subsequently, we assessed the effectiveness of colistin with apigenin in treating animals infected with mcr-1-positive *E. coli*. Firstly, an infection model with *G. mellonella* larvae was constructed to assess the treatment efficacy of the combined strategy mentioned above. The untreated group and colistin-treated group showed a generalized blackening of *G. mellonella* larvae and all survival no more than 3 days (Fig. 5A). The larval survival rate was obviously increased after treatment with the combination regimen, and the survival rate reached 50% after 5 days post infection (Fig. 5A). Moreover, even apigenin treatment group could also achieve 20% protection after 5 days post infection (Fig. 5A). Similarly, the treatment effect shown in the model of mouse peritonitis-sepsis matched the anticipated *in vivo* reaction. In comparison to the untreated group and colistin-treated group, the combination regimen improved the survival rate of mice by 60% and 40%, respectively (Fig. 5B). Furthermore, as compared to colistin alone, the combined regimen significantly decreased the number of bacteria in the mice's liver and spleen, with a decrease in bacterial load of 1.58 and 1.64 Log10, respectively (Fig. 5C). Additionally, the combined therapy strategy group and the apigenin group had significantly lower levels of TNF-α and IL-1β (Fig. 5D). Histopathological analysis of lung tissues showed relatively normal lung tissues in the combination treatment group, whereas inflammatory cell infiltration and congestion were present to varying degrees in both the untreated group and the drug-alone group of apigenin or colistin (Fig. 5E). Compared with the combination and colistin-alone groups in the above results, it has been shown that apigenin could improve the therapeutic efficacy of colistin against animals infected with mcr-1-positive *E. coli*.

## DISCUSSION

The use of antibiotics on a large and illogical scale has resulted in a significant rise of bacterial resistance. World public health security is seriously threatened by AMR among Gram-negative pathogens (27, 28). The widespread spread of plasmid-mediated colistin-resistant mcr-1 genes among bacteria further exacerbates the embarrassing situation that there is no effective drug for MDR infection (7). Therefore, there is an urgent need to find effective treatments, such as *mcr*-1 inhibitors or colistin enhancers, for the treatment of mcr-1-positive bacterial infections. Traditional high-throughput screening requires the screening of massive number of compounds for biological activity, which is cumbersome and costly in terms of experimental and equipment operation (29). In contrast, virtual screening is a computerized simulation and prediction method to screen out potentially biologically active compounds from a large database of compounds, which can greatly accelerate the drug discovery process and reduce the experimental cost and development cycle (30). Here, we combined CADD for high-throughput screening of inhibitors targeting *mcr*-1 protein. In previous studies, flavonoid compounds, including such as honokiol, phloretin, and naringenin, were found to enhance the antimicrobial activity of colistin against mcr-1-positive Gram-negative bacteria (25, 31, 32). Apigenin, discovered using virtual screening, likewise enhanced the antimicrobial activity of colistin. Apigenin is a naturally occurring flavonoid and found in a variety of plants primarily in the form of yellow pigment (33). Here, apigenin performed best in the screening score among flavonoids, and the comparative analysis using online SwissADME tools revealed that apigenin had good physicochemical properties, including compliance with the Lipinski's rule of five and absorption in the gastrointestinal tract, which was consistent with the reference predicting good bioavailability of apigenin and the physicochemical properties analyzed according to Lipinski's rule of five (34). In this study, the combination of apigenin and colistin was effective against MDR bacteria, including mcr-1-positive and mcr-1-negative strains. This suggested that there were several synergistic mechanisms between apigenin and colistin. A series of mechanistic investigations *in vitro* indicated that the enhanced antibacterial effect of apigenin on colistin was mainly attributed to the change thermal stability of the *mcr*-1 protein, the inhibition of mcr-1 expression, bacterial membrane damage, and the induction of bacterial NO and ROS production. Collectively, apigenin was a potential lead chemical that could eventually strengthen the antibacterial activity of colistin against MDR bacteria.

Natural compounds are potential candidates for the development of anti-infective drugs (35, 36). The molecular structure of apigenin determines unique physiological effects and biological properties. Apigenin has antioxidant, anti-inflammatory, and anti-tumor properties (37–40). Compared to other flavonoids (quercetin, kaempferol flavonoids) that are currently known to have anti-inflammatory effects, apigenin has the advantage of lower toxicity and non-mutagenicity (34, 41). Currently, apigenin has some applications in pharmaceutical and food industries. Preclinical studies have shown the benefits of apigenin for skin conditions, and apigenin could be considered a future medicinal product in skin care (42). Apigenin also possesses the role of regulating glucose homeostasis, providing a scientific basis for its development as a functional food (43). Some potential compounds with *mcr*-1 protein inhibitors also exhibit synergistic effects with polymyxin *in vivo* in terms of antibacterial activity (31, 32). Here, animals infected with mcr-1-positive *E. coli* benefited from the combination of colistin and apigenin. Additionally, a bacterial load in the tissues decreased and the survival rate of the treated mice increased noticeably. More meaningfully, apigenin inhibited the production of inflammatory factors and reduced the pathological damage of lung inflammation in animal experiments, all of which suggested that apigenin exerted a good anti-inflammatory effect on animal infection models. Inflammatory response is one of the problematic issues in the later stages of clinical treatment (44); apigenin in combination with antibiotics for the treatment of inflammatory response induced by bacterial infections will be a good choice. However, the poor water solubility and low intestinal absorption of apigenin have led to its low oral bioavailability and low activity *in vivo* and limited its application to a certain extent (45). In this study, we treated the infected mice with gavage administration, which would unavoidably impact the bioavailability of apigenin and lessen therapeutic benefit of the combination. Therefore, further optimization of the efficacy of the combination *in vivo* is still needed. Fortunately, there are several studies to design and develop novel drug delivery systems and dosage forms of apigenin to improve oral bioavailability and make apigenin more suitable for clinical applications (45, 46). In conclusion, without the risk of bacterial resistance, apigenin was a promising lead compound that could greatly enhance the antibacterial efficacy of colistin both *in vivo* and *in vitro*. Studies clearly elucidated the mechanism of action of apigenin in interfering with thermal stabilization of *mcr*-1 protein and aggravating the damage that combination regimens caused to bacterial membranes. The combination of natural plant monomers and antibiotics provided a new strategy to alleviate the serious situation of MDR Gram-negative bacteria and some data for the dual approach of anti-inflammatory and antimicrobial in clinical therapy.

## MATERIALS AND METHODS

### Bacterial strains and reagents

*K. pneumoniae* ZJ02 and *E. coli* ZJ478 were provided by Prof. Yang Wang's team at China Agricultural University. *E. coli* ATCC 25922 was purchased from the American Type Culture Collection (ATCC). *E. coli* CF5-1 and *E. coli* CN3-1 were obtained from chicken manure samples collected in Liaoning, China. Other strains of *E. coli* were isolated from piglet manure samples collected in Liaoning, China. Clinical isolates carrying the mcr-1 gene were identified by amplification with designed primers (Fig. S6). The strains were cultivated dynamically or statically at 37°C after being inoculated into Mueller-Hinton broth (MHB, Qingdao Hope Biotechnology) or MH agar (MHA) plates. The mcr-1 gene from strain *K. pneumoniae* ZJ02 was amplified using the primers listed in reference (31) , and the resultant sequence was then cloned into the expression vector pET28a using the NheI and XhoI restriction sites and maintain the vehicle with 50 µg/mL kanamycin. The target sequence was verified by sequencing. In Dulbecco's Modified Eagle's Medium (DMEM, Gibco), 10% heat-inactivated fetal bovine serum (FBS, Invitrogen), 1% (wt/vol) penicillin-streptomycin, and 1% (wt/vol) sodium pyruvate were added as growth media for Vero cells. Apigenin and colistin were purchased from TargetMol. Apigenin and colistin were dissolved in DMSO and deionized water, respectively.

### Screening method

The three dimensions (3D) structure of *mcr*-1 protein (5GRR) from the RCSB database was utilized. The 3D structure was preliminarily processed by MOE (v. 2009). The structure files of small molecule compounds were obtained from the TargetMol database and the Drug Bank database, and the flow of small molecule pretreatment was constructed by Pipenline Pilot (version 8.5). The Autodock-based software Smina (version 2020) was used to perform the process of virtual screening and score the binding conformation of small molecules to proteins (47). During the screening process, the binding pocket encompassed the entire protein, which allowed ligands to bind freely to the protein, and each combination generated 20 different conformations based on docking scores. Each set of results was docked separately, and the conformation with the highest docking score was output. Smina's built-in scoring function was used for protein and small molecule docking scoring.

### ADMET predictions

The SwissADME platform (www.swissadme.ch) was utilized to compute the pharmacokinetic and ADMET properties of the potential drugs (48) . The prediction of drug-likeness compounds followed Lipinski's five standard rules: molecular weight (MW), numbers of hydrogen bond donors (HBD) and acceptors (HBA), numbers of rotatable bonds (RB), polar surface area (PSA), and lipophilicity (LogP). Furthermore, gastrointestinal absorption and brain penetration were predicted using the brain or intestinal estimated permeation technique (BOILED-Egg), which was incorporated into the SwissADME platform.

### MIC assay

MICs were ascertained using the conventional broth microdilution technique in compliance with the Clinical & Laboratory Standards Institute's standards (49). In a transparent, UV-sterilized 96-well microliter plate (Corning, New York, USA), the medicines were specifically diluted two times in MHB medium. Bacterial suspension with a final concentration of 7.5 × 10^5^ CFU/mL was added. The 96-well microliter plate was incubated at 37°C for 18 hours before the findings were inspected. The MIC value was the lowest antibiotic concentration at which no bacterial growth was observable.

### Checkerboard studies

The fractional inhibitory concentration (FIC) index and the synergistic impact of candidate drugs and antibiotics were computed using the checkerboard technique (50). Firstly, the MHB medium was added to a 96-well plate at 100 µL/well and the drugs were diluted in such a way that the antibiotic was diluted laterally, and the candidate compound was diluted in the other direction. Bacterial cultures grown to the logarithmic phases were adjusted to the 0.5 McFarland turbidity standard, diluted with MHB broth at a factor of 1:100, and added to 96-well plates. The 96-well microliter plate was incubated at 37°C for 18 hours before the findings were inspected. Each of the wells could be scanned for an optical density at 600 nm using a multifunctional Microplate Reader (FLUOstar Omega, Offenburg, Germany). The following formula was used to calculate the FIC values: FIC = (MIC drug A combination/MIC drug A alone) + (MIC drug B combination/MIC drug B alone). FICI values are defined as follows: 1 < FICI < 2, indifference (or no effect); 0.5 < FICI ≤ 1, additivity; and FICI ≥2, antagonism.

### Growth curve

The strain under test was cultured in MH broth medium at 37°C with shaking until an optical density of 0.3 was reached at 600 nm (OD600) (31). Subsequently, the bacterial solution was evenly distributed into Erlenmeyer flasks and unequal amounts of drug were added to each of flasks to form a drug concentration gradient (apigenin 16 µg/mL, apigenin 32 µg/mL, apigenin 64 µg/mL, colistin 4 µg/mL, and apigenin 32 µg/mL + colistin 4 µg/mL). The bacterial growth condition was assessed by measuring the absorbance at 600 nm per hour in Erlenmeyer flasks using a microplate reader (Eppendorf, Hamburg, Germany).

### Time‐dependent killing curve

The potential bactericidal capacity of the apigenin combined with colistin was evaluated by the time-kill curve (51). At a final concentration of 1 × 10^5^ CFU/ml, bacterial cells were cultured for 0, 3, 6, 9, and 12 h with apigenin (32 µg/mL), colistin (4 µg/mL), apigenin (32 µg/mL) in combination with colistin (4 µg/mL), or DMSO, the standard control. Samples from each group were collected at the specific moment above and plated onto MHA solid plates after dilution with PBS. There were three replicates for each dilution. The plates were incubated at 37°C for the entire night. After calculating the CFU, the time-kill curve was drawn.

### Combined disk test

The CDT referred to Kali's method (52). In brief, the strain under test was cultured in MH broth medium at 37°C with shaking until an optical density of 0.1 was reached at 600 nm (OD600). A sterile cotton swab was dipped into the bacterial solution and excess bacterial solution was removed from the cotton swab. Subsequently, it was spread evenly over the entire surface of the MHA plate. Enough bacterial solution was evenly spread throughout the MHA plates. A 10 µg colistin disc was titrated with 10 µL of apigenin (32 µg/mL) at varying doses. The discs were carefully put on the solid agar plate and incubated at 37°C for 24 hours. The diameters of the inhibitory zone were then measured.

### Resistance frequency analysis

After growing *E. coli* ATCC 25922 to the logarithmic phase, the bacteria were diluted 1:1,000 into the MHB medium. The appropriate amount of drug was added. Two groups of drugs were colistin (0.125 µg/ml) and colistin plus apigenin (32 µg/mL). The MIC of the culture was determined after 24 hours at 37°C. At the same time, another culture was diluted to the updated 0.25 × MIC drug for subsequent manipulation. To determine the ratio of the colistin MIC value to the starting MIC value, we carried out this process every day for 30 days (53). Experiments were performed in three biological replicates.

### Molecular dynamics simulation

Molecular dynamics simulations were done by gromacs (version 2018.8), and the initial conformation was generated by the MOE (v. 2009). The complex system was encased in a spherical box filled with water, to which the right amount of Na^+^ was added to maintain the charge balance. The parameters of the composite system were generated using the AMBER force field, and the energy minimization process of the system was carried out. After the complex system had completed a temperature equilibration of 310 K (physiological ambient temperature) and a pressure equilibration of 1 bar, the system proceeded to the final md simulation. In the final MD simulation, which ran in 2 fs of steps size for 10 ns, all hydrogen-containing bonds' motion and long-range electrostatic effects were handled by SHAKE and particle mesh Ewald (PME), respectively. The analysis of the combined free energy of the simulated trajectory file was done by software GMX_MMPBSA (version 1.5.7) (54).

### *mcr*-1 Protein expression and purification

Using the pET28a(+)-based *E. coli* BL21(DE3) expression system, we created the recombinant *mcr*-1 protein and then purified it using Ni-NTA. The *mcr*-1 protein sequence was subcloned into the pET28a(+) plasmid vector by the NheI and XhoI enzyme cleavage sites to form pET28a-mcr-1 plasmid. The successfully ligated plasmids were verified by sequencing. *E. coli* BL21(DE3) cells were transformed intracellularly with the plasmid and then seeded onto MH solid agar plates with 50 µg/mL of kanamycin. The plates were incubated at 37°C for the entire night. Subsequently, the developed single colonies were picked and added to a 1 L liquid medium that contained 50 µg/mL of kanamycin. Cultures were incubated at 37°C until an OD600 nm of 0.6–0.8 was reached. We then added 1 mM IPTG to the cultures and protein expression was stimulated at 16°C for a whole night. To concentrate the microorganisms, we centrifuged the medium for 10 minutes with 10,000 g/min at 4°C. The bacteria were purified on a nickel column following ultrasonic homogenization, and the result was eluted using various imidazole solution concentrations. The product desalted with the eluent was the *mcr*-1 recombinant protein.

### Nano differential scanning fluorimetry

The Nano DSF experiment was conducted using Prometheasn (NanoTemper Technologies, München, Germany) (55). As a sample for subsequent usage, apigenin (32 µg/mL) was incubated with *mcr*-1 (100 µg/mL) in PBS for 20 minutes. Samples were tested through a standard capillary and exposed to an environment between 25°C and 95°C with an increasing rate of 5°C/min. The detector would detect tryptophan fluorescence at 330 nm and 350 nm after 280 nm UV excitation. The parameters of thermal stability were calculated using PR.ThermControl software (version 2.1.5).

### Microscale thermophoresis (MST) assay

The *K*_d_ value of apigenin binding to *mcr*-1 was determined using MST assay (56). The protein was labeled by co-incubating it with RED-NHS second-generation dye for 30 minutes at 25°C in the dark, following the reagent kit's instructions for the assay. For the serial dilution of apigenin, the reaction buffer was PBS buffer (pH = 7.4) with 0.05% Tween-20. Using Monolith NT.115 Pico at moderate MST power and 5% LED/excitation power, we added an equivalent amount of 100 nM of the labeled protein to the dilution. To ensure the reliability of the data, the *K*_d_ value was the result of fitting three sets of data.

### Membrane damage and permeability evaluation

Bacterial suspensions with an OD600 nm of 0.5 were centrifuged and washed two to three times with PBS buffer. The bacteria were resuspended with PBS buffer (OD600 nm of 0.5) and drugs (apigenin 32 µg/mL; colistin 4 µg/mL; apigenin 32 µg/mL + colistin 4 µg/mL) were added. We added 10 µM NPN fluorescent probe (Sigma-Aldrich) or 10 nM PI staining solution (Thermo Fisher Scientific) and incubated it at 37°C for 2 hours in the dark before adding to a black 96-well plate and scanning the fluorescence signal using a multifunctional microplate reader (excitation/emission wavelength: NPN, 350/420 nm; PI, 535/615 nm.). Meanwhile, the above samples were collected for scanning electron microscopy (SU8100, HITACHI, Tokyo, Japan) (57).

### Intracellular nitric oxide

*E. coli* ZJ478 suspensions grown in the logarithmic phase were collected by centrifugation and washed two to three times using PBS buffer to collect the pellet. The bacteria were resuspended with PBS buffer (OD600 nm of 0.5) and drugs (apigenin 32 µg/mL; colistin 4 µg/mL; apigenin 32 µg/mL + colistin 4 µg/mL) or solvent control (containing 0.3% DMSO) were added. The mixture was incubated at 37° for 2 hours. Subsequently. samples were gathered and examined in accordance with the manufacturer's instructions using a nitric oxide test kit (Beyotime, Shanghai, China). The NO relative change fold was obtained based on the ratio of the optical density at 540 nm of each sample to the mean value of the normal group data.

### ROS measurement

The instructions provided by the manufacturer (Beyotime, Shanghai, China) state that 10 × 10^−6^ m 2′,7′-dichlorodihydrofluorescein diacetate (DCFH‐DA) was used to determine ROS. The bacteria were resuspended with PBS buffer (OD600 nm of 0.5) and drugs (apigenin 32 µg/mL; colistin 4 µg/mL; apigenin 32 µg/mL + colistin 4 µg/mL) or solvent control (containing 0.3% DMSO) were added. After 2 hours of drug-treated *E. coli* ZJ478 incubation, fluorescent dyes were added, and the fluorescence intensity was observed at 488 nm for excitation and 525 nm for emission. The intensity of the fluorescence signal of DCFH-DA was proportional to the concentration of bacterial ROS. Data were obtained from three biological replicates.

### Intracellular ATP measurement

The level of intracellular ATP in *E. coli* ZJ478 was evaluated by the Enhanced ATP Assay Kit (Beyotime, Shanghai, China). Bacteria grown in the logarithmic phase were collected, washed, and adjusted to an OD600 of 0.5, and the pellet was resuspended with different concentrations of colistin with or without apigenin. The bacteria were lysed using ATP lysate after 2 hours. The luminescence was detected using a multifunctional microplate reader. The amount of ATP in the sample was calculated from the standard curve of luminescence signal versus the concentration of ATP standard solution.

### Real-time quantitative PCR (qRT-PCR)

*E. coli* ZJ478 suspensions grown in the logarithmic phase (OD600 between 0.6 and 0.8) were collected by centrifugation and washed two to three times using PBS buffer to collect the pellet. The pellet was adjusted to an OD600 of 0.5. Then, drug dilution (apigenin, 32 µg/mL; colistin, 4 µg/mL) or solvent control (containing 0.3% DMSO) was added. The mixture was incubated at 37° for 2 hours. After sample collection, mcr-1 was amplified using a TB Green qPCR Kit (TaKaRa) and 16 sRNA was used as the control gene. The primers involved in the experiment contained two pairs: mcr-1 (for: GGGCCTGCGTATTTTAAGCG; rev: CATAGGCATTGCTGTGCGTC) and 16sRNA (for: CTGGAACTGAGACACGGTCC; rev: GGTGCTTCTTCTGCGGGTAA). A standard amplification procedure using a two-step PCR was performed: 95°C for 30 s, 40 cycles of 95°C for 5 s, and 60°C for 34 s. The program ran in a CFX96 Real-Time PCR Detection System (Bio-Rad). The difference in the expression level of genes was calculated by the 2−ΔΔCt method (58).

### Cell toxicity

Hemolytic toxicity and cytotoxicity of colistin in the presence of apigenin were assessed based on previous studies (59, 60). We treated 4% defibrillated sheep erythrocytes for 1 hour with colistin containing apigenin (32 µg/mL) or without apigenin. We used 0.2% triton-x100 as a positive control. Culture supernatants were collected and assayed for absorbance at OD543. Subsequently, the hemolysis rate was calculated compared to the positive control. The CCK-8 assay (TargetMol, Shanghai, China) was used to detect the cytotoxicity of colistin. Drugs were added to 96-well cell plates containing 1 × 10^5^ cells and incubated at 37° for 24 hours. Finally, the absorbance at 450 nm of the cell cultures was determined. and cytotoxicity was calculated.

### *Galleria mellonella* infection model

Utilizing the *G. mellonella* larval infection model, we assessed the combination protocol's synergistic effects (61). All *G. mellonella* larvae were randomized into four groups (*n* = 10 per group) and injected with 10 µL of infected *E. coli* ZJ478 (1.0 × 10^6^ CFUs) suspension at the right hind gastropod. The PBS solution of the drugs (apigenin group, 2 mg/kg; colistin group, 2 mg/kg; combination group, 2 + 2 mg/kg) was injected from the left hind gastropod 2 hours after infection. Then, the survival of larvae of infection was observed after 120 hours. The control group was injected with PBS buffer with 0.8% DMSO and the untreated group was infected with bacteria without medication.

### Mouse peritonitis infection

Female BALB/C mice were divided into four groups (*n* = 10 per group) and injected intraperitoneally with an *E. coli* ZJ478 suspension at a lethal dose of 2.5 × 10^7^ CFU (62). Mice were infected and treated with drug solutions (apigenin group, 10 mg/kg; colistin group, 10 mg/kg; combination group: 10 mg/kg + 10 mg/kg) by gastric administration 2 hours later. Solvent buffer (containing 0.5% CMC-Na) was given to the control group. After infection, the survival of mice was observed every 24 hours for a total of 7 days. Additional mice infected with 2.0 × 10^6^ CFU of *E. coli* ZJ487 were used for other analyses. Following the necropsy of the mice, we prepared HE-stained paraffin sections for histological examination using the characteristic pathological alterations in the lungs of the various groups. The same microscope was utilized to take all the lung pathological images (Nikon Eclipse C1, Japan), and any potential observed square shapes are likely result from an artifact with the Adobe illustrator CC software (Adobe2019, USA) or microscope. Serum was collected to detect changes in the levels of inflammatory factors. Mouse uncoated ELISA Kits (Invitrogen, California, USA) were used to detect the expression levels of TNF-α and IL-1β following the manufacturer's instructions. The leftover tissues from the liver and spleen were weighed, homogenized, and planted onto plates. After 48 hours, the bacterial load of tissues was determined. The ethics batch number of animal protocols and methods of operation was 2023052.

### Statistical analysis

The mean ± SD values are used to express all experimental results (*n* ≥ 3). The one-way ANOVA with post-hoc Tukey tests was employed in the statistical analysis using GraphPad Prism 9.0.2 (GraphPad Software, San Diego, CA, USA).

## Data Availability

The data presented in this study are available on request from the corresponding author and are available in the supplemental material (Dataset S1).

## SUPPLEMENTAL MATERIAL

Supplemental figures and table spectrum.00341-24-s0001.pdf Fig. S1 to S6; Table S1.

Dataset S1 spectrum.00341-24-s0002.xlsx The data presented in this study.

ASM does not own the copyrights to Supplemental Material that may be linked to, or accessed through, an article. The authors have granted ASM a non-exclusive, world-wide license to publish the Supplemental Material files. Please contact the corresponding author directly for reuse.
