# Supplementary material for: High-throughput screening identification of apigenin that reverses the colistin resistance of mcr-1-positive pathogens
Source: Microbiol Spectr. 2024 Sep 9;12(10):e00341-24. doi: 10.1128/spectrum.00341-24 (PMC11448233; doi:10.1128/spectrum.00341-24)
Supplement: Supplemental figures and table — Fig. S1 to S6; Table S1. [file spectrum.00341-24-s0001.pdf]

Supporting Information

Table S1. Virtual screening of the top 10 scoring natural products.

NO.	Name	CID	Docking Energy (kcal/mol)
9151	Lumefantrine co-artemether	CMP9151	-19.86794
19017	Erythorbic Acid	CMP19017	-19.48618
9086	4-(carbamimidoylsulfanyl)butanenitrile	CMP9086	-17.1343
18579	O6-Benzylguanine	CMP18579	-17.033
18952	4'-Hydroxychalcone	CMP18952	-16.91025
17400	Diclofop-methyl	CMP17400	-16.85131
10198	2,2,2-trifluoroethyl acetate	CMP10198	-16.72373
15897	Apigenin	CMP15897	-16.69238
10604	Fluorofuranylnorprogesterone F-18	CMP10604	-16.62002
1311	2,14,15-trimethyltetracyclo heptadecane-5,14-diol	CMP1311	-16.46232

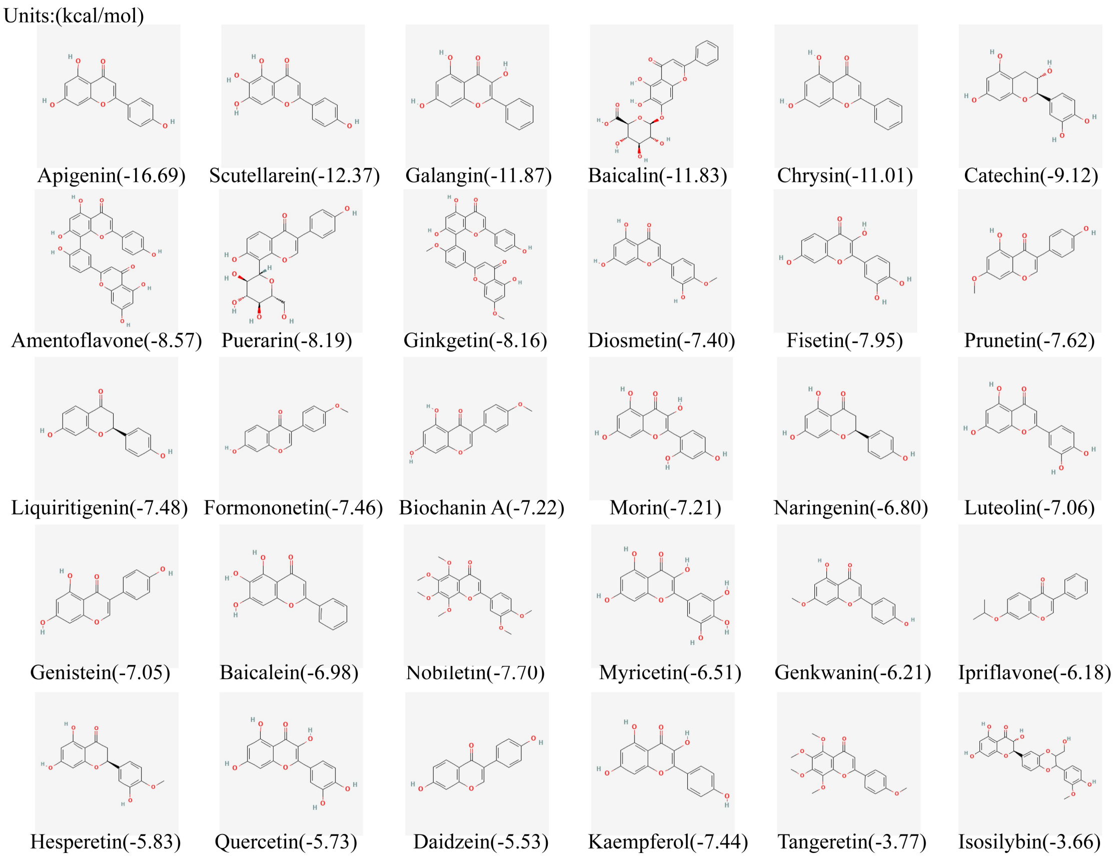

Figure S1. 30 flavonoids and their corresponding docking energy values.

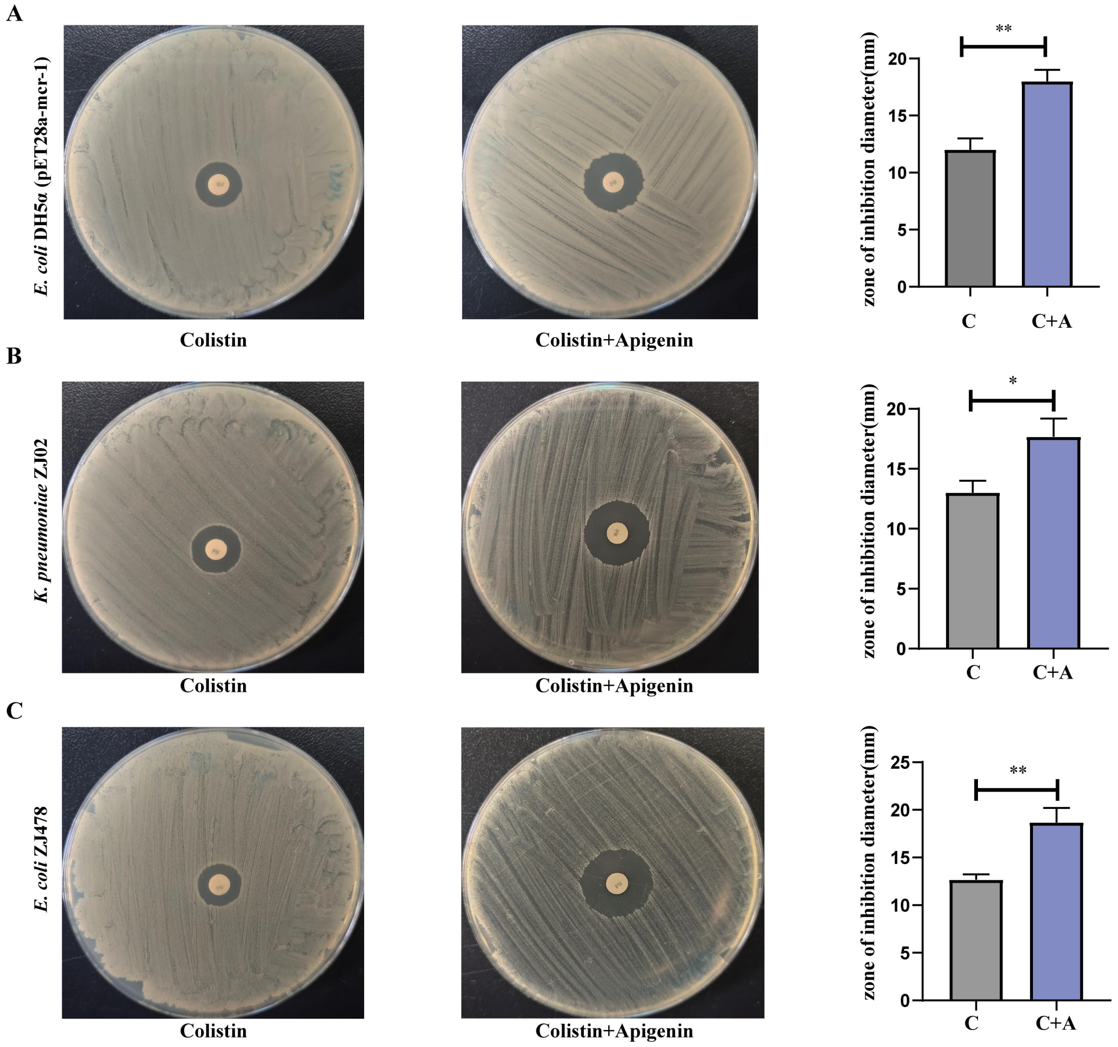

Figure S2. Zones of inhibition surrounding colistin(10μg) disks supplemented with 32μg/ml apigenin on MHA plates and the corresponding inhibition circle diameter statistics. (A) *E. coli* DH5α (mcr-1); (B) *K. pneumoniae* ZJ02; (C) *E. coli* ZJ478. Data was obtained in three biological replicates.

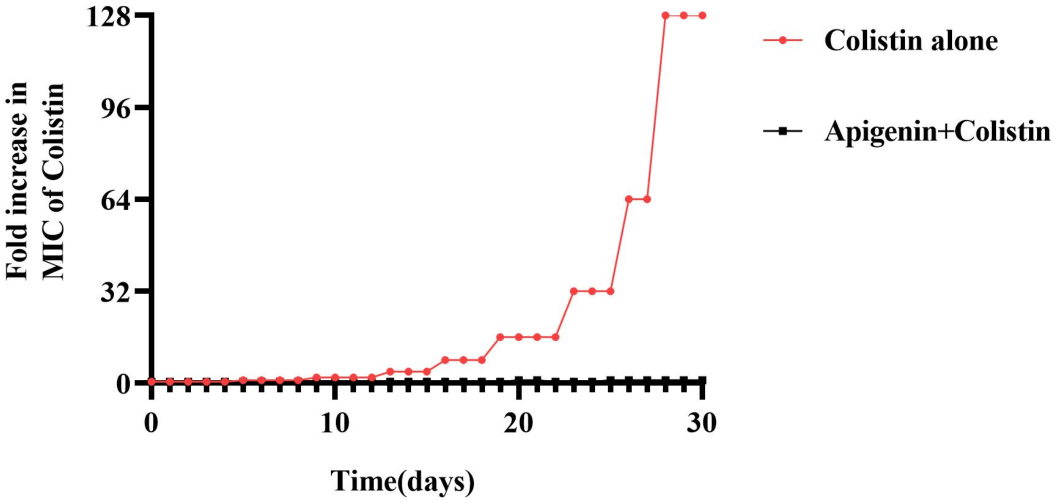

Figure S3. Resistance development of *E. coli* ATCC25922 was detected by serial passaging with colistin. Drugs concentration: colistin (0.125 μg/mL) and colistin plus apigenin (32μg/mL). The process was carried out every day for 30 days

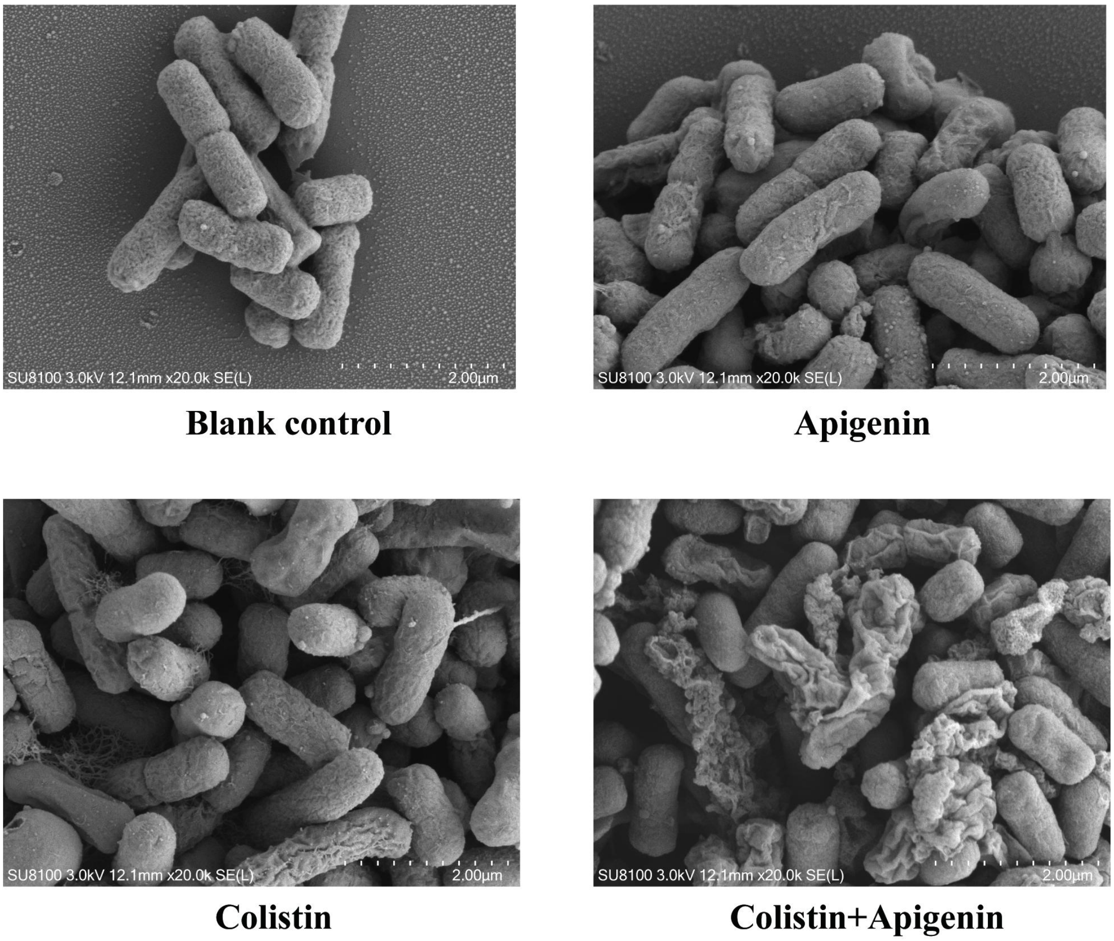

Figure S4. SEM images (×20,000) of *E. coli* ZJ478 with drugs. Apigenin 32 μg/mL;

colistin 4 μg/mL; apigenin 32 μg/mL + colistin 4 μg/mL.

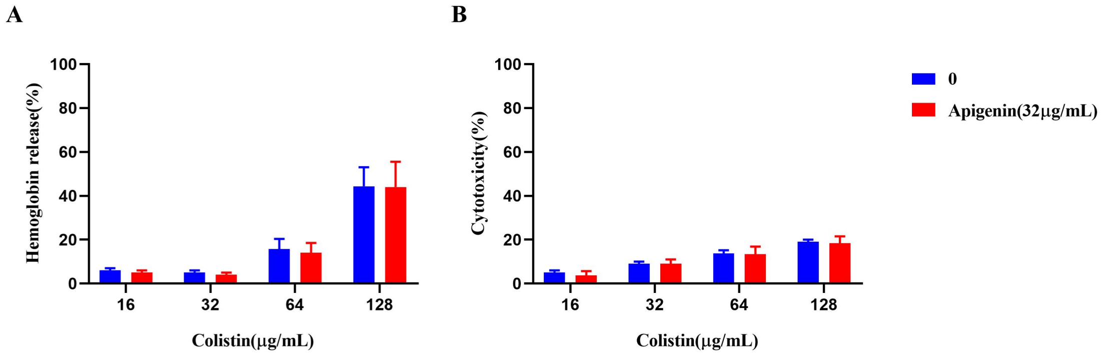

Figure S5. (A) Hemolytic activity of colistin to RBCs with or without apigenin; (B) The cytotoxicity of colistin on Vero cells with or without apigenin. The hemolysis rate was calculated compared to the positive control. The CCK-8 assay was used to detect the cytotoxicity of colistin. Data was obtained in three biological replicates.

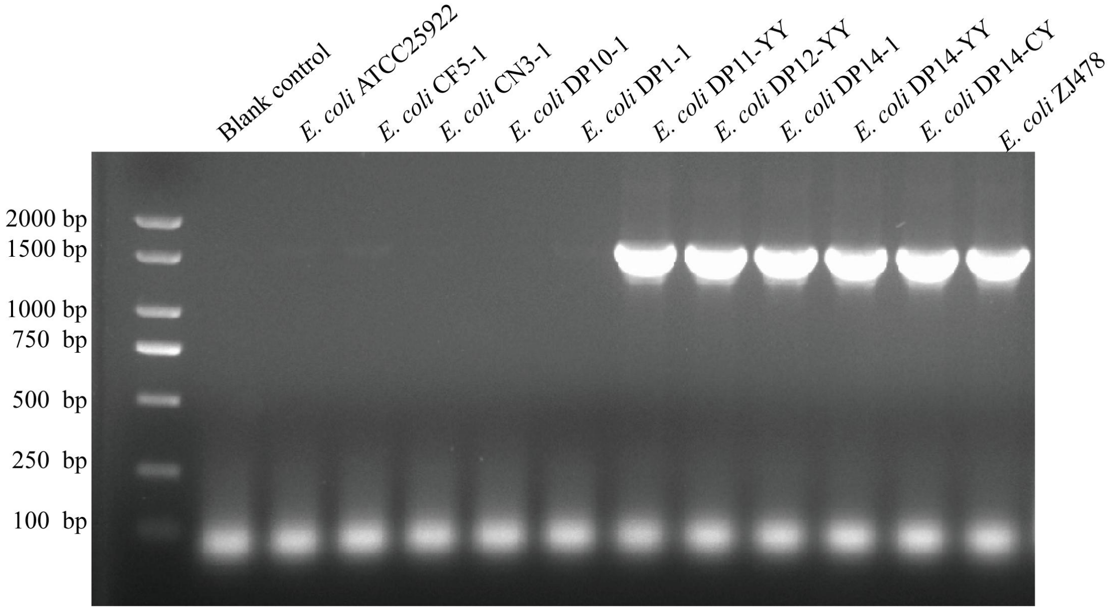

Figure S6. The mcr-1 gene of the clinical strain was amplified, and the sequences of the amplification primers were as follows:

for:ATGATGCAGCATACTTCTGTGTGGT,

rev:TCAGCGGATGAATGCGGTGCGGTCT.
